# An Open-Source AI Framework for the Analysis of Single Cells in Whole-Slide Images with a Note on CD276 in Glioblastoma

**DOI:** 10.3390/cancers14143441

**Published:** 2022-07-15

**Authors:** Islam Alzoubi, Guoqing Bao, Rong Zhang, Christina Loh, Yuqi Zheng, Svetlana Cherepanoff, Gary Gracie, Maggie Lee, Michael Kuligowski, Kimberley L. Alexander, Michael E. Buckland, Xiuying Wang, Manuel B. Graeber

**Affiliations:** 1 School of Computer Science, The University of Sydney, J12/1 Cleveland St, Sydney, NSW 2008, Australia; ialz9547@uni.sydney.edu.au (I.A.); guoqing.bao@sydney.edu.au (G.B.); rzha7694@uni.sydney.edu.au (R.Z.); 2 Ken Parker Brain Tumour Research Laboratories, Brain and Mind Centre, Faculty of Medicine and Health, University of Sydney, Camperdown, NSW 2050, Australia; cloh4889@uni.sydney.edu.au (C.L.); yzhe8012@uni.sydney.edu.au (Y.Z.); 3 St Vincent’s Hospital, Victoria Street, Darlinghurst, NSW 2010, Australia; svetlana.cherepanoff@svha.org.au (S.C.); gary.gracie@svha.org.au (G.G.); 4 Department of Neuropathology, RPA Hospital and Brain and Mind Centre, Faculty of Medicine and Health, The University of Sydney, Sydney, NSW 2006, Australia; maggie.lee@sydney.edu.au (M.L.); kim.alexander@lh.org.au (K.L.A.); michael.buckland@sydney.edu.au (M.E.B.); 5 Sydney Microscopy and Microanalysis, The University of Sydney, Sydney, NSW 2006, Australia; michael.kuligowski@sydney.edu.au; 6 Neurosurgery Department, Chris O’Brien Lifehouse, Camperdown, NSW 2050, Australia; 7 School of Medical Sciences, Faculty of Medicine and Health, University of Sydney, Camperdown, NSW 2050, Australia

**Keywords:** artificial intelligence, bifocal convolutional neural network (BCNN), CD276, image fusion, image segmentation, PathoFusion framework

## Abstract

**Simple Summary:**

We present a workflow for the artificial intelligence (AI)-based profiling of individual cells in whole-slide scans of histological tissue sections. We have extended the PathoFusion framework to automatically detect, count and identify (segment) individual immunochemically labelled cells. We used CD276, a protein of interest in glioblastoma, as a marker and focused our analysis on a subpopulation of labelled cells which may represent glioblastoma stem cells (GCS). Additional studies on the identity of these interesting cells are required.

**Abstract:**

Routine examination of entire histological slides at cellular resolution poses a significant if not insurmountable challenge to human observers. However, high-resolution data such as the cellular distribution of proteins in tissues, e.g., those obtained following immunochemical staining, are highly desirable. Our present study extends the applicability of the PathoFusion framework to the cellular level. We illustrate our approach using the detection of CD276 immunoreactive cells in glioblastoma as an example. Following automatic identification by means of PathoFusion’s bifocal convolutional neural network (BCNN) model, individual cells are automatically profiled and counted. Only discriminable cells selected through data filtering and thresholding were segmented for cell-level analysis. Subsequently, we converted the detection signals into the corresponding heatmaps visualizing the distribution of the detected cells in entire whole-slide images of adjacent H&E-stained sections using the Discrete Wavelet Transform (DWT). Our results demonstrate that PathoFusion is capable of autonomously detecting and counting individual immunochemically labelled cells with a high prediction performance of 0.992 AUC and 97.7% accuracy. The data can be used for whole-slide cross-modality analyses, e.g., relationships between immunochemical signals and anaplastic histological features. PathoFusion has the potential to be applied to additional problems that seek to correlate heterogeneous data streams and to serve as a clinically applicable, weakly supervised system for histological image analyses in (neuro)pathology.

## 1. Introduction

We have previously reported on our open-source AI framework termed PathoFusion [1] which allows the marking, training, and automated recognition of histological features in whole-slide images (WSIs) of diagnostic human tissue sections. Our present study extended the applicability of the PathoFusion framework to the cellular level. The new method is particularly attractive for the analysis of immunochemical stains and may improve diagnostic efficacy.

Manual microscopic analysis of entire histological slides at cellular resolution is very time-consuming. Depending on the type of tissue and on the labelling results obtained, this task may become prohibitively complex and expensive for human observers [2]. Therefore, an automated system for the immunochemical microscopic analysis of histological WSIs at 40× primary magnification would be very desirable.

With the development of digital pathology, many researchers expect that AI, especially deep learning (DL), will be able to assist in the workup of diagnostically difficult entities, as well as research into their causes. Thus, AI guidance may play a critical role not only for clinical tumour detection (e.g., neuroimaging, biomarkers) but also for more accurate and timely tissue diagnostics and prognostication. The main challenge when extracting features from histopathological image is the identification of morphological characteristics that are associated with disease-typical cellular and tissue alterations. The adoption of DL methods has caused a decline in interest in feature extraction methods, as the representation and decision boundaries can be learned in a single optimization process and these methods have therefore become dominant over traditional machine learning approaches. The work of Malon et al. [3] was the first to use deep learning methods for histological images analyses. Their proposed method used recognition of deep features by a convolutional neural network (CNN) to train a support vector machine (SVM) classifier to locate mitotic nuclei in histological images. Zerhouni et al. [4] used a wide residual convolutional neural network to detect mitotic figures in histological images. Li et al. [5] proposed a weakly supervised deep segmentation network for mitosis detection. Their method is based on expanding the weak label of a mitosis centroid to a novel label consisting of concentric circles, and a concentric loss function is then used to train the network to perform the mitosis segmentation. Sheikh et al. [6] proposed a multiscale and multifeature network model that fuses multiresolution feature maps at different layers. This proposed model learns different scale features to account for global and textural cellular features at the same time.

**Figure 1:**
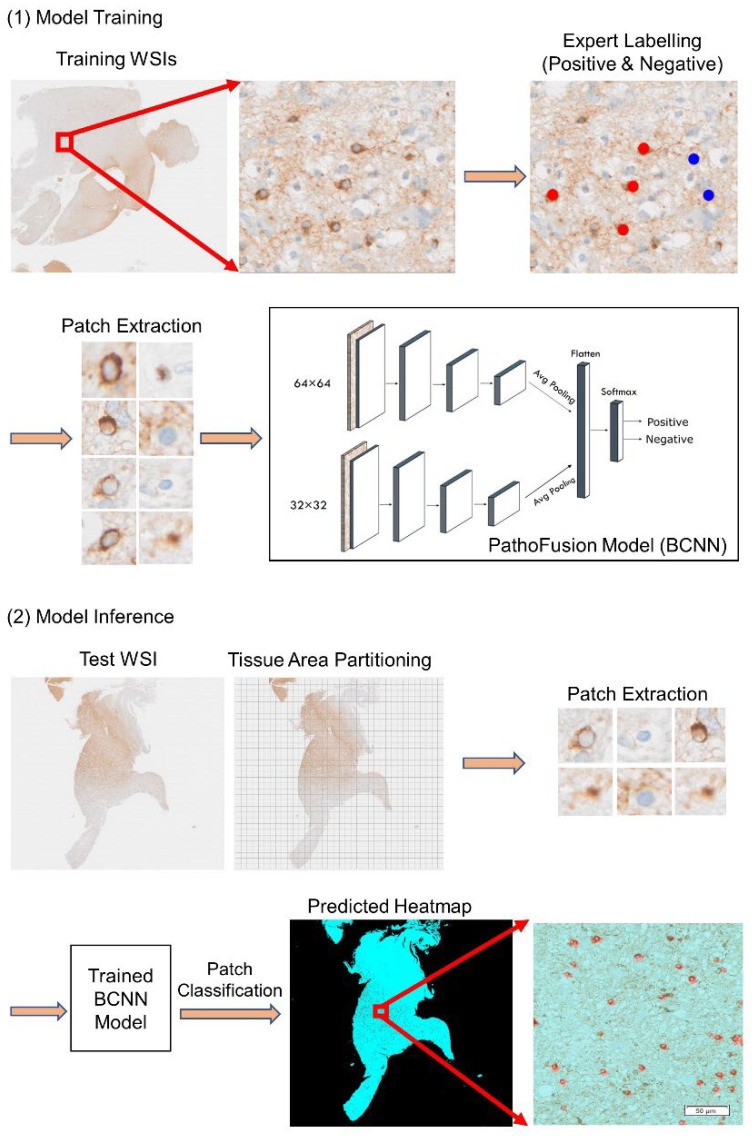
Illustration of the extended PathoFusion framework used for the analysis of cell profiles in whole-slide images (WSI). Slides were subjected to immunochemistry for CD276, followed by brief hemalum counterstaining. (**1**) Model training: Patches were extracted from a WSI in line with specialist (consultant neuropathologist) annotations and passed to the bifocal convolutional network (BCNN). (**2**) Model inference: Following partitioning of the test image (the grid size shown is not to scale and is for illustration purposes only), extracted patches are provided to the trained BCNN model for classification and the prediction results are converted into the corresponding heatmaps (the pseudo-colour red marks recognized cells). Scale bar: microns.

**Figure 2:**
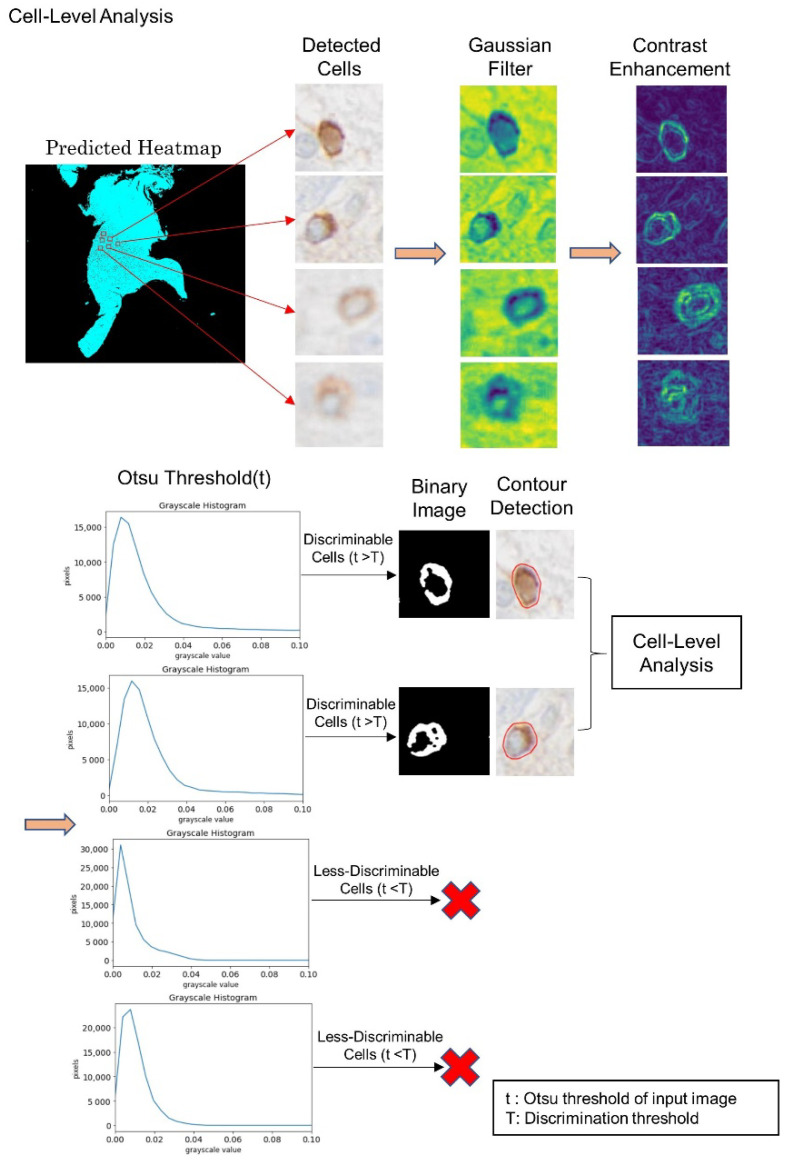
Cell-level analysis. Image patches containing cells identified by the BCNN are then processed for cell-level analysis. Filtering and thresholding are followed by edge and contour detection. An image is considered discriminable if the minimum acceptance value for the Otsu threshold is reached. It can then be segmented using edge and contour detection.

This study serves to provide a comprehensive and effective workflow for the artificial intelligence (AI)-based identification of individual cells in histological tissue sections and represents an important next step towards an automated diagnostic system that will also integrate genomic data. We have used glioblastoma biopsies and CD276 as an immunochemical marker for testing the extended PathoFusion system. CD276 (B7-H3) is an immune checkpoint molecule of special interest in cancer research [7,8,9]. On the basis of detailed microscopic analyses of CD276-labelled glioblastoma biopsies, we have identified a subpopulation of cells which, because of their strong perinuclear immunoreactivity, we have tentatively termed “halo cells” (Figure 1 and Figure 2). Interestingly, the number of “halo cells” varied significantly across biopsies, stimulating questions concerning their role in glioblastoma. According to recently published literature, it seems possible that “halo cells” represent a type of CD276-expressing cancer stem cells [10], i.e., glioblastoma stem cells (GSCs).

As the old term glioblastoma multiforme emphasized, glioblastomas are extremely heterogenous morphologically. This applies not only to the macroscopic visual and neuroimaging but also to the histological and even to the cellular and subcellular levels. Not only the tumour cells themselves but also other cells, e.g., microglia and macrophages that are found in these tumours in high numbers, show a great variety of phenotypes. In addition, there is great intratumoral heterogeneity regarding the distribution of the various cell types in glioblastoma, which may include all the cell types present in normal brain tissue that has been infiltrated by the tumour, as well as peripheral immune cells. While the original PathoFusion framework is capable of learning to detect histological features, the improved cellular resolution of the extended framework now allows tumour analyses in fine detail at the cellular and potentially subcellular level, including the mapping of markers for cancer stem cells.

## 2. Materials and Methods

### 2.1. Dataset

Whole-slide scans of WHO Grade 4 glioma samples, provided by the Australian Genomics and Clinical Outcomes of Glioma (AGOG) tissue bank, were used for this study (University of Sydney Human Ethics Committee Project number 2016/027). Paraffin sections were stained with H&E and scanned at 40× magnification using an Olympus VS−120 scanner. Adjacent sections were processed for CD276 immunochemistry. Immunochemistry for CD276 was carried out on an automated Ventana system. The SP265 antibody (Abcam, Melbourne, Australia) directed against C-terminal CD276 was used. Whole-slide scans of glioblastoma samples from a second independent cohort provided by the Sydney Brain Tumour Bank, part of the Neuropathology Tumour and Tissue Bank, Royal Prince Alfred Hospital (Royal Prince Alfred Hospital Ethics Committee Project number 2019/ETH07282), were used for validation purposes.

Immunoreactive cells were individually marked using the PathoFusion platform, which integrates a labelling website [1]. CD276-positive cells were marked under supervision of a consultant neuropathologist (MBG). Histopathological features (including normal brain tissue and blood vessels, infiltrating tumour, microvascular proliferation, and geographic and palisading necrosis) in adjacent H&E-stained tissue sections were annotated following WHO criteria and correlated by PathoFusion as described earlier [1]. In total, 31,947 cells were manually marked using the website on 8 immunochemically labelled whole-slide scans, which were each split into 4 image tiles to handle the very large file sizes. These cases were selected from the same cohort used previously [1]. The second independent cohort (also described above) was used for validation. In addition, 18,767 regions covering various morphological features in negative areas (no “halo cells” present) were marked resulting in a total of 50,714 marking coordinates. For each one of the 50,714 individually marked coordinates, a pair of image patches was extracted (sizes of 32 × 32 and 64 × 64 pixels, respectively) from the 32 slide image tiles showing immunochemical labelling that had been used for testing (4 image tiles representing 1 WSI per case). Training and testing data were extracted from different cases and there was no overlap between them, i.e., 36,734 paired image patches from the total number of cases were used for training and cross-validation, and 14,005 paired image patches were used for testing.

### 2.2. The Framework

Our methodology for cell profiling in WSIs of immunochemically stained tissue sections consists of two main steps: detection and segmentation of individual cells. The extended PathoFusion framework is illustrated in Figure 1: (i) patches of two different sizes with their coordinates based on ground truth labelling are extracted from immunochemically labelled WSIs to allow the BCNN to learn both the cellular information and the surrounding tissue structures; (ii) the BCNN model [11] is trained as a feature extractor and predictor; (iii) at the prediction stage, the detection results from the whole-slide tissue sections are converted into corresponding heatmaps; (iv) this is followed by filtering and thresholding to segment only the discriminable cells by means of edge and contour detection for further cell-level analysis.

Our aim was to apply PathoFusion at the cellular level for the purpose of profiling individual immunolabelled cells in WSIs. We used CD276 immunoreactive “halo cells” as an example to lay the groundwork for the identification of pathological changes at the cellular level. The performance of the detection process is highly dependent on the features extracted from the images. In our case, analogous to the diagnostic algorithm a microscopist in (neuro)pathology may be using, a specific tissue area of interest (i.e., containing a cell) and its surroundings (the tissue context) were both considered when patches of two different sizes (image tiles of two different sizes) were processed by the CNN. This bifocal design significantly improved the performance of the detection process, as demonstrated earlier [1]. The patches measuring 32 × 32 pixels placed a narrow focus on cell-related characteristics and were extracted as contiguous overlapping patches from the WSIs with a stride of 10 pixels. In addition, we extracted overlapping 64 × 64 patches with a stride of 10 pixels that contained each of the smaller patches and their surroundings, placing a wider focus on the tissue area of interest. All extracted patches were given a label indicating either the presence or absence of “halo cell(s)”.

### 2.3. The BCNN Model

Our BCNN model is more efficient as a feature extractor and detector than other deep learning models [11]. The BCNN has two input paths and consists of two convolutional sub-nets, one feature concatenation module and a classification layer. Usually, the training of a deep neural network requires many training images to avoid over-fitting. However, our BCNN model requires far less training [11]. To improve model generalization, image augmentation including rotation, contrast and sharpness adjustments was randomly applied to the training dataset. At the evaluation and testing stage, paired image patches were extracted from the upper left to bottom right of the whole-slide test images with a stride of 10 pixels. Each patch pair was classified by the trained BCNN model and assigned to one of two categories, positive or negative for CD276 expression by “halo cells”. The prediction results were converted into the corresponding WSI heatmaps using pseudo-colours illustrating the distribution of the detected cells across entire tissue sections.

### 2.4. Filtering and Thresholding

To distinguish clearly recognizable (“discriminable”) CD276 immunoreactive cells from weak (“less discriminable”) positivity, a binarization algorithm (Otsu thresholding) was used to post-process the prediction results of the BCNN model. This was preceded by filtering.

Only patches predicted to contain “halo cells” were allowed to pass through the filtering and thresholding process. First, these patches were converted into grayscale images. Next, a Gaussian filter was applied to remove noise caused by various internal and external factors. In a Gaussian filter, noise is spotted with the surrounding information, so the average value of the neighbouring pixels replaces the noisy pixel present in the image based on a Gaussian distribution, reinforcing the smoothness of the respective images. After de-noising of the images, contrast enhancement was performed by enhancing the variation in the pixel intensity in each neighbourhood. Accordingly, each pixel value of the resulting image indicates the contrast intensity in the nearby pixels.

For automatic thresholding, we used Otsu’s method [12] to determine the suitability of the detected cells for further analysis. Our thresholding method determines the minimum intensity value of the “halo cell” pixels using Otsu’s algorithm. Specially, we utilized images cropped around the cells that had been detected by the BCNN model [11]. Otsu’s algorithm [12] assumes that the input image contains two classes of pixels that follow a bimodal distribution (foreground and background pixels, respectively) and sets an optimum threshold such that the intra-class variance is minimized and the inter-class variance is maximized. In our cropped images, “halo cell” pixels have the highest intensity values, whereas there is not much information (immunoreactivity) around the cells. The intensity level returned by the Otsu algorithm refers to the minimum intensity of a cell’s pixels. Therefore, if the Otsu algorithm returns a high value, this indicates that the intensity of a cell’s pixels in the input image is high (a discriminable cell). In contrast, if the algorithm returns a small value, this mean that the cell’s pixels do not differ much from the background (a less discriminable cell). After determining the Otsu thresholds (intensity levels) for all patches, we needed to find a sufficiently general minimum value for the pixel intensity (T) of the “halo cells” (discriminable threshold) that could be used as the criterion for distinguishing cells that have been successfully detected. We also needed to minimize inter-class variances among the detected “halo cell” images which could be used in our proposed Equation (1) after confirmation of its validity on our cases. If a patch’s Otsu threshold surpassed the discriminable threshold, T, as calculated by Equation (1), then that patch was deemed to be discriminable. The discriminable threshold can vary from WSI to WSI, as the antibody labelling intensity can differ between WSIs. Figure 2 illustrates the filtering and thresholding process in detail.

The formula for finding the minimum acceptance value for the Otsu threshold (discriminable threshold) of “halo cell” images is given by Equation (1):

(1) $$T=\frac{\sum \sigma^{2}}{N}-t_{min}$$

where *T* is the minimum acceptance value for the Otsu threshold of an input image, $\sum \sigma^{2}$ is the sum of the Otsu threshold values of all input images, *N* is the number of input images and $t_{min}$ is the lowest threshold found among all input patches.

### 2.5. Edge and Contour Detection

As a part of our post-processing workflow, edge and contour detection were used for further segmentation and more precise measurements of the discriminable “halo cells” in the relevant patches. Edge and contour detection facilitates the location of the cells of interest in an input image, determining their cell area, perimeter, compactness and minimum (min) pixel intensity. Edge detection refers to the process of identifying and locating points in the input image where the intensity changes greatly; it is very high on the edges. A contour is defined as the closed curve joining all points that have the same intensity, corresponding to the shapes of the objects in the image. Thresholding is used to binarize the discriminable cell images highlighting the cells of interest in white on a black background and allowing the contour detection algorithm to work. Thresholding turns the border of the cells in the image completely white, with all pixels having the same intensity range as shown in Figure 2 (binary images). In our “halo cell” images, the colour changes typically happen at the boundaries of the cells. This allows the identification of edges simply by observing the change in colour. The corresponding intensity class is not constant, but the rate of change in intensity is highest at the edges. The contour detection algorithm can sketch the borders of the cells from the white pixels and find the contour points around the perimeter of the cells. Case-level features were obtained by calculating the mean for cell area, perimeter, compactness and minimum pixel intensity.

### 2.6. Discrete Wavelet Transform for Image Fusion

Image fusion permits precise co-localisation of classical diagnostic features defined in H&E-stained sections with immunochemical results, thus increasing the amount of information that can be obtained from biopsy specimens and aiding the diagnostician in their clinical assessment. In other words, the fusion of medical images can generate higher quality content by combining complementary information from multiple sources into a single image.

**Figure 3:**
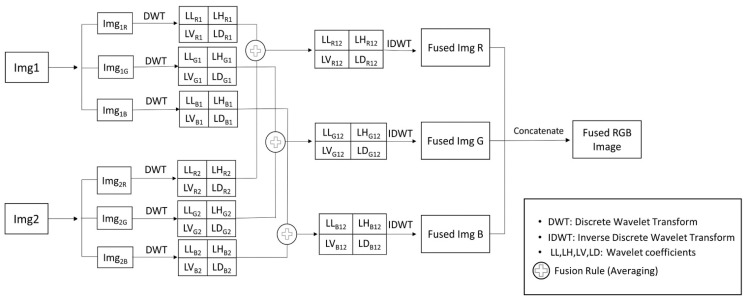
Image fusion schematic illustrating our use of the Discrete Wavelet Transform, with pixel averaging as the fusion rule on the RGB channels of input images.

In order to fuse images of H&E-stained sections with sections immunolabelled for CD276, the Discrete Wavelet Transform (DWT) [13] was implemented by using the average intensities of corresponding pixels from both the input H&E and CD276 (“halo cell”) heatmaps. Specifically, single-level discrete wavelet decomposition was applied to the RGB channels of the H&E and CD276 heatmaps, resulting in a four-component image consisting of approximate, horizontal, vertical and diagonal wavelet coefficients for each channel (Figure 3). The wavelet coefficients were then fused using averaging, where the average value of each corresponding pixel from both input heatmaps was assigned to the corresponding pixel of the output image (Equation (2)). Once all bands of each RGB channel had been transformed, an inverse wavelet transform was applied to the fused components to create the fused multispectral image for each channel. Finally, concatenation of the RGB channel images was performed.

(2) $$Fused_{Img}\left(x,y\right)=\left(Img1\left(x,y\right)+Img2\left(x,y\right)\right)/2$$

where *Img*1 (*x*, *y*) and *Img*2 (*x*, *y*) are the input images (the H&E and CD276/“halo cell” heatmaps, respectively), and $Fused_{Img}$ is the resulting fused image.

## 3. Results

“Halo cells” were detected using our BCNN model. The performance of the model was evaluated and compared with that of other deep learning models. Heatmaps showing the distribution of the detected cells in entire whole-slide images were then created. Measurements concerning individual “halo cells” were statistically analysed. Fusion maps of “halo cells” and H&E heatmaps revealed the exact tissue distribution of “halo cells“. Whole-slide cross-modality analyses of “halo cells” were performed to obtain information on their spatial relationship to anaplastic histological features. The individual analysis steps are detailed in the following sections.

### 3.1. Performance Evaluation

Our BCNN model was used for automated detection of “halo cells” in WSIs [11]. Performance was evaluated using the following criteria: accuracy, precision, specificity and F1-score. In addition, a confusion matrix was created.

The BCNN model, the subnet structure of the BCNN model and Resnet−50 [14] were used with identical parameters (learning rate, weight decay, momentum, patch size and number of epochs). The stochastic gradient descent (SGD) algorithm was used to train the models. The learning rate was set to 0.0005, gradually decreasing while training; the batch size was 32 with 70 epochs; the momentum was 0.9 and the weight decay 0.005. The patching strategy described in the Methods section yielded 50,714 pairs of image patches in the training set following extraction from the 32 image tiles, representing eight whole-slide scans with individual patches measuring 64 × 64 and 32 × 32 pixels. To introduce generalization, we used data augmentation techniques while training.

For our experiments (inference on test cases; Figure 1, lower panel), between 10 and 35 million patches per case were fed into the BCNN. Specifically, patches of the different sizes (32 × 32 and 64 × 64 pixels) were passed through the two input channels of the BCNN model, providing two categories of patch-level output, including the information as to whether “halo cells” were present. For clarity, the two categories were then assigned two different pseudo-colours. Outcomes that indicated the presence of “halo cells” then passed the filtering and thresholding scheme to select only discriminable cells for further analysis (Figure 2). Our BCNN model identified 12,211 “halo cells” in our test WSI, while the filtering and thresholding process discarded 4263 cell profiles following calculation of the minimum acceptance (discrimination) threshold, 0.020; the image threshold values ranged from 0.010 to 0.059. Thus, a total of 7948 “halo cells” were kept in the case of our test WSI.

**Figure 4:**
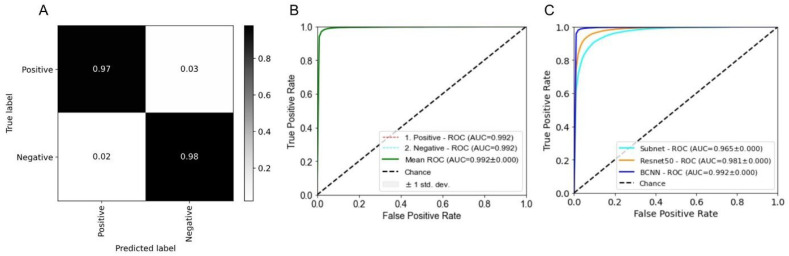
(**A**) Confusion matrix for “halo cell” detection. (**B**) Area under the curve (AUC) and receiver operating characteristic (ROC) analysis of “halo cell” detection by our BCNN model. (**C**) Comparison of ROC/AUC performance of the BCNN with that of two other deep learning models.

PathoFusion achieved a high prediction performance for “halo cells” (AUC of 0.992), as shown in Figure 4B and as apparent from the confusion matrix in Figure 4A. For comparison, two other deep learning methods were used: the subnet structure of our BCNN model and the popular state-of-the-art ResNet−50 [14]. Figure 4C shows the comparison of the ROC curves and AUC scores of the three models. Taken together, our experimental results demonstrate that the PathoFusion model (BCNN) achieved superior results to ResNet−50 and the subnet structure in terms of accuracy, precision, specificity and F1-score (97.7%), as shown in Table 1.

### 3.2. Visualization

**Figure 5:**
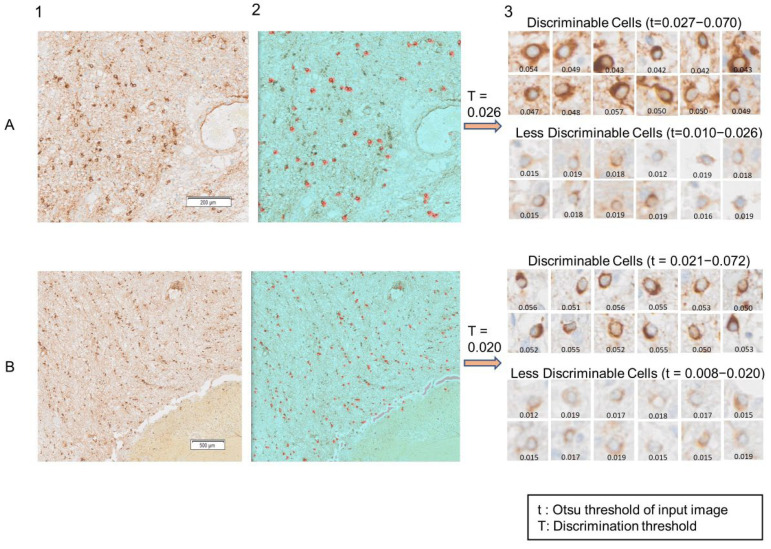
(**A**,**B**) The prediction results generated by the BCNN model for two independent cases. (**1**) Low-magnification view of an area showing “halo cells”; (**2**) the corresponding predicted heatmap revealing the detected “halo cells” (in red). (**3**) Visualizations of the results of the filtering and thresholding process: (**3A**,**3B**) upper panel: discriminable cells that returned Otsu values exceeding the discrimination threshold, (**3A**,**3B**) lower panel: Less discriminable cells whose Otsu value fell below the discrimination threshold. Scale bar: microns.

PathoFusion’s proficiency in patch-level recognition [1] formed the basis for the automated analysis of entire histological sections in our study. As demonstrated in Figure 5, our methodological improvements allow PathoFusion to be used at the single-cell level. The BCNN model reliably identified “halo cells” (marked red on the turquoise heatmap; Figure 5) after iteratively learning from patches extracted on the basis of ground truth labels. Furthermore, our BCNN model demonstrated the ability to identify “halo cell” profiles efficiently and consistently in WSIs, a task that would be extremely time-consuming and exhausting to achieve for a human observer who also lacks the ability to refer to an accurately defined threshold of detection.

### 3.3. Statistical Analysis

“Halo cells” are amongst the most visible and most discrete objects in our immunochemically labelled images, which is why they were selected for testing. Typically, they are relatively dark brown and have a round to elliptical shape, but some may appear slightly irregular due to variations in their staining density, which can affect their measurement. Therefore, we developed an edge and contour detection algorithm and applied it to all discriminable (“halo cell”) patches to analyse them using four of the most visually distinctive properties: area, perimeter, compactness, and minimum pixel intensity, which were then averaged to generate case-level features (Table 2).

The area of each cell refers to the total number of pixels occupied by each cell, and the unit is pixels. Perimeter refers to the number of pixels on the circumference of the cell; its unit is also pixels. Area and perimeter were calculated by OpenCV after finding the contour of each cell. The minimum pixel intensity of each cell was calculated by Otsu thresholding. Compactness is the ratio between a cell’s area and its perimeter, calculated according to Equation (3) [15]:

(3) $$Compactness=\frac{{\left(perimeter\right)}^{2}}{4\pi \;\times \;area}$$

Interestingly, we found evidence that the number of “halo cells” may correlate with patient survival (data not shown). This finding requires a detailed follow-up, as it could be of future use as a prognostic factor in patients with glioblastoma. However, a detailed analysis of this problem goes well beyond the scope of the present study.

### 3.4. Fusion of Bimodal Histological Images

**Figure 6:**
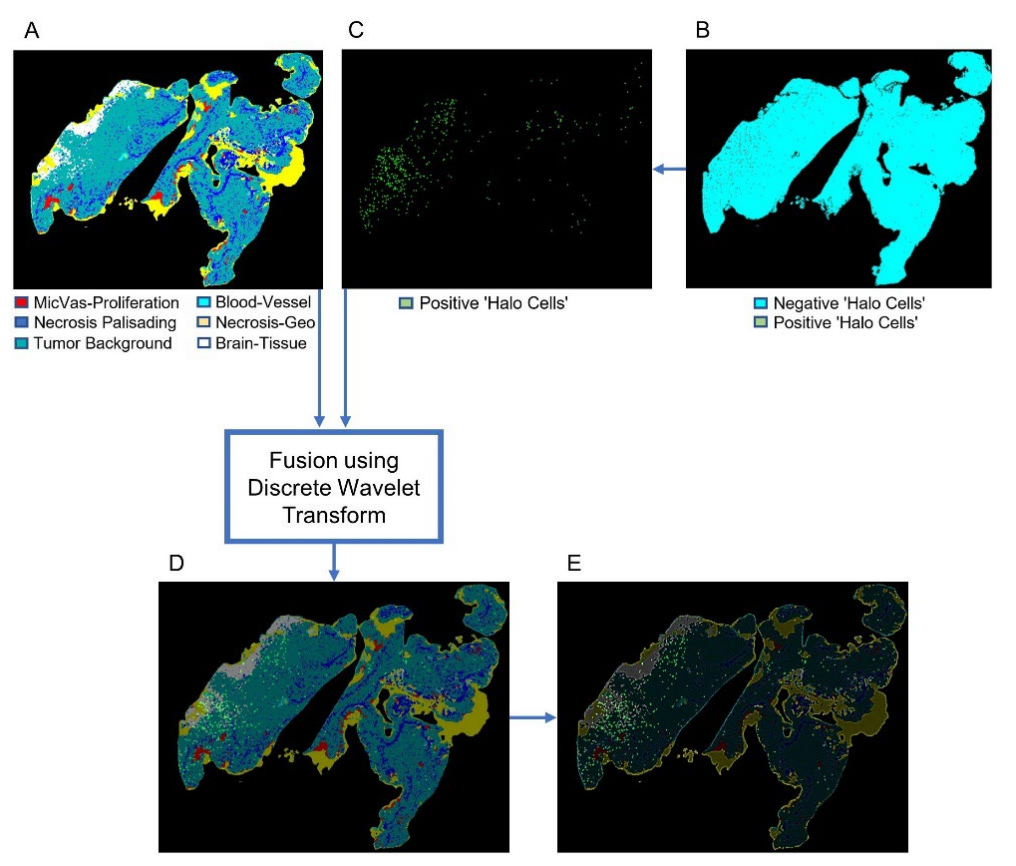
Image fusion using Discrete Wavelet Transform. (**A**) Original heatmap showing histological features. (**B**) Heatmap showing cell-level features (“halo cells” and negative areas). (**C**) Thresholding, registration and alignment, resulting in a heatmap showing only the cells of interest. (**D**) The Discrete Wavelet Transform was used to fuse heatmaps (**A**,**C**); (**E**) shows the same image as (**D**) but with greater brightness to emphasize the location of “halo cells” (bright dots). Whole slide images are shown.

The current study extended the versatility of PathoFusion. Pixel averaging of the fused H&E and “halo cell” heatmaps by the DWT [13] allowed detailed comparisons of cases while preserving anatomical references (Figure 6). Many different techniques have been proposed for medical image fusion, such as intensity, frequency and Laplacian pyramid-based methods. Some of these techniques are based on spatial frequency analyses, while others are based on spatial orientation selectivity. DWT allows both frequency and spatial analyses, and is capable of detecting all features contained in a signal [13]. This method effectively preserved both the coarse and fine details of our input images. DWT divided the input image into sub-images, which were then fused (Figure 6).

As shown in Figure 6B,C, we applied thresholding to the “halo cell” heatmaps to select the cells of interest, followed by image registration and alignment with the H&E diagnostic feature map (Figure 6A). Different points in the same locations on both heatmaps were then matched by a registration algorithm [16]. Figure 6D shows the H&E heatmap (cf. [1]), which was taken as the reference image with which the “halo cell” heatmap, shown in Figure 6E, was fused.

Finally, the correlation of immunolabelled “halo cells” with diagnostic morphological features [1] was visualized in the bimodal WSIs through image fusion of the corresponding predicted heatmaps. There were differences in the number of “halo cells” among cases. The density of “halo cells” in relation to the diagnostic morphological features is summarized in Table 3.

## 4. Discussion

With the development of digital imaging in pathology, computer-assisted diagnostic (CAD) algorithms have become popular. CAD systems can now complement diagnoses made by a pathologist, facilitating decision-making and improving the efficiency of the diagnostic process [17]. Early CAD systems were based on conventional learning algorithms, where much of the effort was spent on feature extraction based on expert domain knowledge, followed by the application of traditional classification models, including random forests and support vector machines [18,19,20,21,22,23,24]. However, some engineered machine-learning features are of limited use in biomedical applications. Wang et al. [25] proposed a framework in which integrated radiological and histopathological data analyses including molecular, cellular and texture levels were used to predict isocitrate dehydrogenase genotypes, a clinically significant diagnostic classifier in diffuse glioma.

Deep learning systems, especially CNNs, are capable of extracting morphological features automatically following suitable training [26]. Since it is impractical to feed a CNN directly with images of enormous size such as WSIs, many workers in the field have adopted a patch-based approach to extract features while preserving essential information for detection and classification tasks. Bejnordi et al. [27] presented a context-aware stacked CNN for classification and used CNN training on high-pixel-resolution patches to extract cellular level features, followed by the application of a second CNN. Wang et al. [28] proposed an automated cell type classification pipeline to convert a pathology image into a spatial map of the cells contained therein and then used it to extract features related to the tumour microenvironment. Based on these features, the authors developed an image feature-based prognostic model. Failmezger et al. [29] used a computational pathology pipeline for the classification of cell types in H&E-stained sections. Spatial interactions between cell types were computed using a graph-based algorithm (topological tumour graphs, TTG). In [30], Mousavi et al. proposed a method for automated brain tumour grading based on spatial domain analysis. These authors introduced a method for cell segmentation, and a customized operation of spatial and morphological filters to identify microvascular proliferation, followed by a hierarchical decision tree for low- and high-grade glioma classification. Hatipoglu et al. [31] proposed a cell segmentation approach for histopathological images that used deep learning algorithms and analyses spatial relationships by collecting cellular and extracellular samples from histopathological images through windowing for small patches of varying sizes. Chandradevan et al. [32] reported successful detection and classification based on a fast R-CNN built on a resnet101 fully convolutional network.

In this study, we automatically detected, visualized and segmented cells of interest, i.e., “halo cells”, in the given WSIs. We have taken advantage of the capability of PathoFusion [1] to identify pathological changes characteristic of glioblastoma and to correlate the location of CD276 immunoreactive cells. This was achieved by converting the detection signals into corresponding heatmaps visualizing the distribution of the detected cells in entire WSIs. Our new method achieved 97.7% accuracy.

Our finding that an increased presence of “halo cells” appears to be correlated with shorter patient survival is intriguing. A detailed analysis of this finding goes beyond the scope of this study. However, a discussion of some properties of CD276 and how they can be investigated in future using the technology introduced in this study seems appropriate. CD276 expression by the tumour vasculature is well established [33]. The BCNN could be trained to recognize immunoreactive endothelial cells, and thePathoFusion framework could be used to quantify abnormal blood vessels. In addition, a comprehensive AI-guided correlation between CD276 expression and diagnostic features of malignant glioma, e.g., the different types of necrosis, should be feasible. However, other aspects of CD276 expression are of greater interest in the present context. Wang et al. and Liu et al. [7,10] have previously demonstrated the upregulation of CD276 in cancer stem cells, and CD276 expression has been shown to be associated with GSC self-renewal [34]. Therefore, a detailed analysis of the tissue distribution of “halo cells” and their correlation with other key molecules in glioblastoma would be important and is now feasible. For instance, Sun et al. [35] demonstrated a positive correlation between CD276 and stemness markers (CD133/PROM1, NGFR, TYH1, SOX2), and Johnston et al. pointed out the correlation with immune modulators (i.e., IFNGR1, IFNGR2, TNFRSF1A and TNFSRF1B) and self-renewal genes, including VAX2, SOX21 and CITED1 [34]. CD276 has also been shown to be involved in the epithelial–mesenchymal transition [7]. Importantly, CD276 expressed by tumour cells may affect the differentiation of tumour-associated macrophages [36], and CD276 is also expressed by at least some macrophages in GBM tissue [37]. The extent of this expression requires further scrutiny, given the heterogeneity of macrophage marker expression in glioblastoma (unpublished data). Therefore, analysing macrophage marker expression in adjacent tissue sections followed by image fusion is an attractive way forward.

## 5. Conclusions

PathoFusion’s BCNN model makes it now possible to automatically identify and count individual immunochemically labelled cells in routine paraffin sections. Our experimental results indicate that PathoFusion is potentially also suitable for the detection of subcellular structures. It could work as part of a general and clinically applicable (weakly supervised) system for cross-modality analyses in (neuro)pathology.

The known advantages of PathoFusion, such as effective trainability based on a comparatively small number of cases and flexible use of consultant time during training, can thus be applied to the growing number of immunochemical markers which require extensive diagnostic evaluation.

## Figures and Tables

**Table 1:** Performance evaluation of different deep learning models.

Model	Accuracy	Precision	Recall	F1-Score
BCNN	97.7%	97.7%	97.7%	97.7%
Subnet (BCNN)	90.0%	90.0%	90.0%	89.8%
Resnet−50	94.0%	94.0%	94.0%	94.0%

**Table 2:** Cell-level features of the cases used.

Heading	Feature	Value Range
	Number of detected “halo cells”	100–15,000 cells
	Density of “halo cells”	0.0003–0.045 cells/mm^2^
Cell-level features	Cell area	7000–11,000 pixels
	Cell perimeter	450–800 pixels
	“Halo cell” pixel intensity	0.026–0.070
	Compactness	2.5–4.5

**Table 3:** Density of “halo cells” in relation to diagnostic morphological features in adjacent H&E-stained sections.

Morphological Features	Number of Halo Cells	Density—Number of Halo Cells/mm^2^
Normal blood vessels	481	0.051
Normal brain tissue	554	0.036
Geographic necrosis	2247	0.047
Viable tumour tissue	7045	0.034
Palisading necrosis	248	0.031
Microvascular proliferation	1636	0.036

## Data Availability

Sample datasets for image patches are openly available in https://github.com/guoqingbao/Pathofusion/tree/master/data, accessed on 15 July 2022.
